# Mitigating Sports-Related Concussions in Adolescent Athletes: A Systematic Review and Meta-Analysis of Communication and Education Interventions

**DOI:** 10.3389/phrs.2025.1608153

**Published:** 2025-05-16

**Authors:** Giovanna Pedroni, Yara Barrense-Dias, Michael von Rhein, Oliver Gruebner, Chantal Kuske, Barbara Goeggel Simonetti, Marta Fadda, Anne-Linda Camerini

**Affiliations:** ^1^ Faculty of Biomedical Sciences, Università della Svizzera italiana, Lugano, Switzerland; ^2^ Department of Epidemiology and Health Systems, Université de Lausanne, Lausanne, Switzerland; ^3^ Child Development Center, University Children’s Hospital Zurich, Zurich, Switzerland; ^4^ Health Sciences and Medicine, University of Lucerne, Lucerne, Switzerland; ^5^ Institute of Pediatrics of Southern Switzerland, Ente Ospedaliero Cantonale (EOC), Bellinzona, Switzerland

**Keywords:** concussion, sport, education, communication, intervention

## Abstract

**Objectives:**

Sports-related concussions (SRCs) in adolescents may pose severe health consequences, which underscores the importance of adequate prevention, early detection, and management. This systematic literature review and meta-analysis aims to synthesize findings from studies evaluating SRC communication and education interventions targeting adolescent athletes and/or their caregivers.

**Methods:**

We included original, peer-reviewed studies published in English between 2014 and 2024. For studies reporting on comparable outcomes, we conducted a meta-analysis based on mean differences between pre- and post-assessment.

**Results:**

Of 2,974 identified records, 22 were included. Most SRC communication and education interventions focused on the North American context, targeted adolescent athletes, and combined digital and in-person communication Interventions were generally more effective in increasing knowledge or awareness than in shifting attitudes or reporting behaviors. The quality of the included studies varied considerably. The studies tended to be short-term and effects appeared independent of the target group, features, or outcome assessments.

**Conclusion:**

Given the findings of this review and meta-analysis, future interventions should aim towards long-lasting attitude change fostering intentions to and behaviours promoting the prevention and early detection of SRCs.

## Introduction

Mild Traumatic Brain Injury (mTBI), commonly called concussion, is a subcategory of TBI and one of the most frequent injuries in children and adolescents [1, 2]. Based on data from the United States (US), children have a lifetime estimate of a parent-reported TBI diagnosis of 2.5% [3]. Furthermore, in the US, studies show that 20% of adolescent and 28% of college athletes have suffered from at least one concussion in their lifetime (compared to about 10% of non-athlete adolescents) [4]. Data on TBI in children and adolescents in the European context are not systematically collected. For all ages and TBI severities, crude incidence rates range from 47 per 100,000 to 694 per 100,000 per year [5]. However, concussions often go unreported and undiagnosed given their invisible nature, the lack of knowledge surrounding concussion, and the athletes’ reluctance to report an incidence [6].

Concussions have been associated with problems in personality, mood, and cognition [7] as well as the onset of neurodegenerative diseases in later life [8]. However, the current literature does not strongly support long-term negative outcomes [9], especially in amateur athletes and youth with prior concussions [10, 11].

Concussions in childhood mostly occur during falls or other types of accidents at home or in school settings [12]. A particular risk context is sports, especially contact sports such as hockey, rugby, football, or basketball. Sports-related concussions (SRCs) may pose severe outcomes not only because of the nature of the sport itself, but also because of the potential social and competitive pressure leading to under-reporting or ignoring a suspected SRC. Players may fear disappointing their team if they stop practising or playing because of a suspected SRC [10, 11, 13–17].

Preventive measures proved to be indispensable in reducing the incidence of SRCs, and research in this field continues to increase [18]. Prevention can be categorized according to four different strategies [19]. First, *technical measures* comprise the use of safety equipment, such as helmets, which can reduce the likelihood, severity and duration of SRC symptoms [20–22]. Second, *legislative measures* include rules and policies, such as the prohibition of body-checking in hockey games involving children and adolescents [23, 24]. In soccer, the prohibition of hitting the ball with the head proved to reduce the frequency of concussion [25]. A third strategy involves *monetary measures*, i.e., taxes and fines as punishment if rules have not been respected, or monetary incentives in form of discounts or rewards for the use of safety equipment [26, 27]. The fourth prevention strategy includes *communication measures*, typically in form of health communication campaigns or education interventions, with the aim to educate people towards healthier behaviours, using various channels and resources. In addition, many interventions of this kind extend beyond direct influence on the population, seeking to shape public policy and the social environment to support behaviour change [28–30].

Currently, the literature on SRC prevention deals with various strategies, rarely focusing on a single approach [18, 31]. This diversification reflects the recognition that the joint effects of different prevention measures can outweigh those of a single strategy. Moreover, individual measures often need to be more easily distinguishable from each other. This poses a challenge for any effort aiming to tease out specific measures’ effectiveness.

Many health communication campaigns and education interventions have targeted coaches of adolescent athletes, e.g., by providing information on ensuring safe training and managing SRCs [32, 33]. Coaches are a preferred target because they typically decide how to implement prevention measures and manage SRCs during training or play. However, not all activities during training and play can be witnessed by the coach, and coaches may underestimate or decide not to follow up with a suspected SRC because they hold negative attitudes towards and inaccurate beliefs about SRC and its management [34]. Therefore, it is pivotal that adolescent athletes and their parents and/or caregivers are knowledgeable about the severity and symptoms of and the necessary actions to deal with a suspected SRC [35]. This knowledge is especially important considering that symptoms often manifest hours after the injury occurred [33]. To this end, several SRC communication campaigns to date have addressed children, adolescents, and their caregivers, such as the prominent *Heads Up* campaign conducted by the Centers for Disease Control and Prevention (CDC) or the recently developed FIFA Concussion Toolkit [36–38]. In brief, CDC’s *Heads Up* campaign is in place since 2003 and provides essential resources for concussion prevention and education through materials like online trainings, videos, and public service announcements, reaching millions of people and educating healthcare providers, coaches, and communities to improve concussion awareness and management [39]. In addition, the recently developed FIFA Concussion Toolkit provides resources for concussion awareness and management in football. It addresses professional players, coaches, medical staff, and the grassroots community. The toolkit uses a mix of posters, educational videos, presentations, and social media resources. Customization options are available for affiliated associations to include players and national brands. The toolkit emphasizes the need to recognize concussion symptoms, seek early medical attention, and ensure a safe return to play by following global guidelines for concussion awareness [38].

Like the two showcased examples, SRC communication and education measures aim to increase awareness of and provide information about how to prevent and manage SRCs. According to established health behaviour theories such as the Theory of Planned Behaviour [40, 41] or the Health Belief Model [42], better awareness and knowledge of SRC contribute to an adequate risk assessment and more positive beliefs and attitudes towards SRC prevention, which are necessary to guide SRC prevention behaviour [43].

However, the scientific literature has evidenced a clear intention-behaviour gap in different health domains [44, 45] including SRC prevention. In other words, the translation of SRC awareness and knowledge into preventive behaviours often remains elusive [46, 47]. Several other factors come into play, such as the sports culture that sometimes downplays the importance of SRCs, and the social and competitive pressure not to stop training or playing despite potential risks. Addressing this gap requires an integrative approach, including interventions that not only inform about the seriousness of SRCs, but also address the psychological and social barriers that prevent the adoption of preventive behaviours [33, 47].

Considering this research gap, we aimed to systematically review SRC communication and education interventions focusing on strategies to promote the awareness, knowledge, attitudes, and behaviours needed to effectively prevent and detect SRCs in adolescent athletes and/or their caregivers – particularly in the context of sport.

## Methods

We performed a preregistered systematic review (PROSPERO Registration Nr. CRD42023485725) following the updated Preferred Reporting Items for Systematic Reviews and Meta-Analyses (PRISMA) statement [48].

### Search Strategy

In December 2023, we systematically searched the following six academic databases: Communication and Mass Media Complete, Psychology and Behavioral Sciences Collection, and APA PsycINFO, Web of Science and MEDLINE, and PubMed/MEDLINE.

The search strategy was based on the PICOS scheme [49]. As population, we included adolescents between 10 and 24 years of age using the definition of adolescence proposed by Sawer and collegues [50], which is based on evidence that the brain develops beyond teenage years as well as on socio-cultural changes over the last decades that let to prolonged dependency on parents in developed countries with access to higher education. As intervention, we considered all studies reporting on the development, implementation and/or evaluation of a health communication strategy or education intervention. We did not specify the comparison as we considered all types of interventions, i.e., not only those using a randomized controlled trial (RCT) design. Furthermore, we did not specify the outcome as we were interested in a wide range of outcomes, including, among others, awareness, knowledge, attitudes, intentions, and behaviours. Concerning the setting, we limited our search to publications focusing on children and adolescents practicing team sports. We specified a second setting by focusing on mTBI. After an initial manual screening of the identified records, we excluded recurring words unrelated to our research question. The complete list of keywords and their combination with Boolean operators is reported in Supplementary Table S1. We limited our search to title and abstract to increase precision and reduce the number of irrelevant records, particularly given the interdisciplinary nature of SRC-related terminology. Subsequently, we hand-searched the reference list of previously published systematic reviews and meta-analyses for additional eligible articles.

### Screening, Data Extraction, and Statistics

We imported all identified records into Zotero to remove duplicates and non-peer-reviewed records (e.g., theses, conference proceedings). We screened the remaining records in two stages: during the screening of titles and abstracts, two reviewers (R1 and R2 - mutually blinded) independently reviewed all records following predefined eligibility criteria. We only included studies that: (1) reported on concussion education for (2) healthy adolescents and/or their caregivers, (3) focusing on a team sports context and (4) head trauma as injury. We focused on team sports as players in teams may be particularly pressured by social norms and conflicting interests that may be specifically addressed in SRC interventions. Furthermore, we only included (5) quantitative original research studies that were (6) published in a peer-reviewed journal (7) in English and (8) within the last 10 years (2014–2023). We decided to focus on the last decade to collect insights into SRC interventions and their effectiveness based on the most recent studies, reflecting current practices, theoretical frameworks, and public awareness related to SRCs. Moreover, from the year 2014 onward, an exponential increase in publications on concussion knowledge was observed.

We calculated the Cohen’s kappa statistic to assess the inter-coder reliability [51]. A third reviewer (R3) screened the records with discordant decisions from the first two reviewers. Next, R1 carried out a full-text screening of the included records and extracted data about the study design, sample, intervention features, theoretical background, and results. R2 did the same for three randomly selected articles to ensure good reliability during data extraction, and the extracted information were compared. There were no discordant points.

In addition to the preregistered protocol, we included a meta-analysis to pool the effect sizes of comparable intervention outcomes across the included studies. We used the major package in the open-access Jamovi statistical platform. We included only studies with the following comparable outcomes: knowledge, attitudes, and reporting behaviour. We used the standardized mean difference as the outcome measure. For studies reporting standard errors or confidence intervals, we calculated the standard deviations. We compared post-intervention to pre-intervention measures considering both the pre-post assessment of single-arm intervention studies and the intervention group pre-post assessment of RCTs. When more than one post-assessment was conducted, we considered the last assessment to account for potential intervention fade out effects (e.g., “Fup2” = second follow-up assessment). For each analysis, a random-effects model was fitted to the data. We estimated the amount of heterogeneity (i.e., tau [2]) using the restricted maximum-likelihood estimator [52]. In addition to the estimate of tau [2], we inspected the I^2^ statistic [53]. Several moderators were considered individually to investigate possible contributors to heterogeneity: gender (% males), age category (younger adolescents aged 11 to 15 and older adolescents aged 16 to 20 based on the distinction proposed by Sawyer et al. [50], caregivers), intervention modality (in-person, online), and time between pre- and post-assessment (in days). The rank correlation test and Egger’s regression test, using the standard error of the observed outcomes as predictors, were used to check for funnel plot asymmetry.

### Quality Assessment

We assessed the studies’ quality following the CONSORT 2010 checklist of information to include when reporting a randomised trial. We adapted the checklist because not all criteria were applicable to each of the study designs, e.g., single-arm interventions (Supplementary Table S2). For each criterion, we assigned 0 (not met), 0.5 (partially met), 1 (fully met), and “NA” if the criterion was not applicable. We classified the included studies taking into consideration their highest possible score (i.e., excluding the criteria that were not applicable) as follows: poor (less than 50% of all criteria met), acceptable (50%–75% of all criteria met), good (more than 75% of all criteria met). R1 carried out the quality assessment. R2 did the same for six randomly selected articles and the assessments were compared. There were no discordant points.

## Results

**FIGURE 1:**
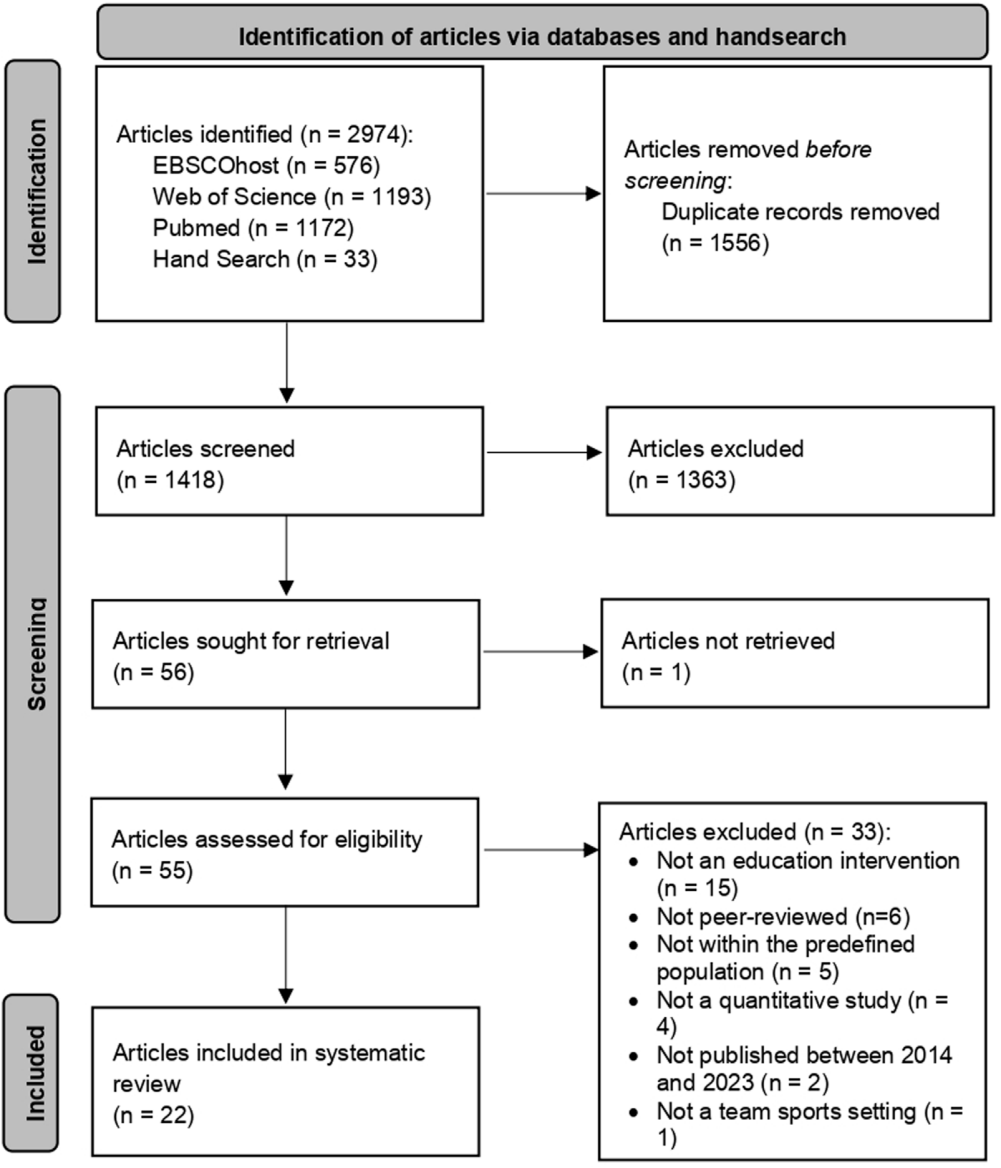
PRISMA flowchart (Lugano, Switzerland, 2024).

Our database search resulted in 2,941 records. We complemented it with a hand search resulting in 33 additional records, for a total of 2,974 (Figure 1). After duplicate removal (n = 1,556), we obtained 1,418 records for title and abstract screening. Intercoder reliability among R1 and R2 was moderate (Cohen’s kappa = 0.699) [51]. During the full-text screening of all retained articles (n = 55; 1 text could not be retrieved), 33 articles were removed for the following reasons: not an education intervention (n = 15), not peer-reviewed (n = 6), not within the predefined population (n = 5), not a quantitative study (n = 4), not published between 2014 and 2023 (n = 2), and not a team sports setting (n = 1). For the remaining 22 articles, reporting each on one study, we performed a quality assessment (Supplementary Table S3) and extracted the relevant data for synthesis (Table 1).

**TABLE 1:** Summary of study characteristics (Lugano, Switzerland).

	Author Publication Year	Gender (%male), Age (M, SD)	Age category (younger adolescents, older adolescents, caregivers)	Sample size	Country	Sports type	Study Design; Time btw pre and post-assessment (days)	Intervention type, location and modality	Main outcome concept	Main results	Direction of effect (+, -, =)	Theoretical background
1	Caron 2018	100%; M_age_ = 15.94 ± 0.34	Older adolescents	35	Canada	Basketball, ice-hockey	Pre-Post Experiment; 28–56	Four interactive oral presentations (30 Min per presentation); In-person; Offline	CK, CA	Post-intervention CK scores were significantly higher than their pre-intervention scores. No significant changes in CA scores post-intervention	CK: + CA: =	Knowledge to action cycle
2	Cranmer 2021	50.67%; M_age_: n.a	Caregivers	600	United States	Football	Experiment; 1	Concussion Intervention Model (CIM). 5 different perspectives from which the narratives were shared; Alone, Offline	other	Concussion Intervention Model is effective (for caregivers) in encouraging intentions to have conversations about SRC with children	CIM: +	Elaboration likelihood model
3	Cusimano 2014	% n.a.; M_age_ = 11.6	Younger adolescents	267	Canada	Hockey	Pre-Post Experiment; 56	Educational video; In-person; Digital	CK, CS	A single viewing of a field hockey educational video can immediately improve concussion knowledge, however, this effect is transient because it is lost after 2 months of follow-up	Immediately: + After 2 mth: =	None
4	Eagles 2016	100%; M_age_ = 14.52	Younger adolescents	57	Canada	Hockey	Prospective cohort study; 1–60	Concussion-U educational program; In-person; Mixed	other	A Concussion-U educational program resulted in an immediate improvement in knowledge and attitude toward concussion among hockey players. The increase in knowledge was maintained at long-term follow-up, but attitude improvement did not occur	CK: + CA: =	Theory of planned behaviour and the Integrated behaviour model
5	Echlin 2014	53.12%; M_age_ = 13.5	Younger adolescents	299	Canada	Physical education students	Longitudinal Study; 7	Interactive electronic teaching program; In-person; Digital	CK	The interactive electronic teaching program (ETP) module led to a greater increase in concussion knowledge between the 2 test sessions	ETP: +	Theory of planned behaviour
6	Elliott 2016	47.32%; M_age_ = 12.5	Younger adolescents	858	United States	Physical education students	Pre-Post Experiment; 1	Workshop based on HSYS program; In-person; Offline	CK	A significant improvement occurred between the mean scores on concussion knowledge of pre- and post-workshop tests. Even in the long term, there seems to be retention of knowledge (+7% from baseline)	HSYS: +	None
7	Glang 2015	Youth: 55.90% Caregivers: 38.50%; M_age_: n.a	Younger adolescents and caregivers	25	United States	Physical education students	Pre-Post Experiment: 27	Brain 101: The Concussion Playbook - web-based program including sports concussion training and resources for educators, coaches, students, and parents; Alone; Digital	CK	Brain 101 can help schools create a comprehensive concussion management program	Brain 101: +	Theory of planned behaviour
8	Hunt 2015	61.76%; M_age_ = 14.78 ± 1.38	Younger adolescents	68	United States	High school athletes	Pre-Post Experiment; 1	Educational video; In-person; Digital	CK	After the educational video, participants’ symptom knowledge and previous concussions reported increased	Video: +	None
9	Kantorski 2020	Youth: 53.30%; Rage = 10-18 Caregivers: 46.67%	Younger adolescents	Youth: 14 Caregivers: 7	United States	Student athletes	Experimental study; 1	Mobile app to increase concussion knowledge, symptoms, management; Alone; Digital	other	“Rebound: Beating Concussions” is an effective learning tool, providing 1) greater knowledge of the symptoms of a concussion, 2) greater knowledge and understanding of what a concussion is and how it can be treated, and 3) dissipation of commonly held misconceptions	Rebound: Beating Concussions: +	None
10	Kroshus2015	100%; M_age_ = 19.15 ± 0.85	Older adolescents	256	United States	Hockey	Pre-Post Experiment; 1–28	Two videos and one informational handout; Alone; Mixed	CS, CK, CA	Across all 3 conditions, perceived underreporting norms increased 1 month after intervention	Educational intervention: -	Theory of planned behaviour
11	Kroshus 2023	73.7%; A_Range_ = 9–14	Younger adolescents	339	United States	Soccer	Pre-Post Experiment; 35–70	Pre-Game Safety Huddles: in-person messages about the importance of reporting symptoms of a suspected concussion; In-person; Offline	CS	Pre-Game Safety Huddles increase the expected likelihood of athletes reporting concussion symptoms	Pre-Game Safety Huddles: +	None
12	Kurowski 2015	77.42%; A_Range_ = 13–18	Older adolescents	496	United States	Football, soccer, wrestling, and basketball	Pre-Post Experiment; 1	Educational lecture; In-person; Offline	CS	A didactic-based preseason concussion education likely has minimal benefits: other factors besides knowledge are likely influencing student-athlete concussion reporting behaviour	Immediately: + Over time: =	Theory of planned behaviour
13	Labiste 2021	center% n.a.; M_age_: n.a	Caregivers	254 Caregivers	United States	Baseball	Pre-Post Experiment; 1–365	Educational intervention called PitchSafe (=short video that contains examples of baseball-related head injuries, signs and symptoms, testimony); In-person; Mixed	CK, CA, CS	There are significant improvements both in knowledge and attitude of concussion. This happens immediately following the intervention and remains stable over the period of a year	CA, CK: +	None
14	Macdonald 2016	100%; M_age_: n.a	Caregivers	47	United States	Contact sport	Pre-Post Experiment; 1	Educational program based on a training module developed by the CDC originally designed for educational sessions with coaches; In-person; Mixed	other	One-time educational intervention is not sufficient to move many parents to be proactive regarding sports-related concussions	Educational program: =	None
15	Manasse-Cohick 2014	% n.a.; M_age_: n.a	Older adolescents	160	United States	High school football players’	Pre-Post Experiment; 14	Short video, followed by a PowerPoint presentation; In-person; Digital	CK, CA	No significant changes in the Concussion Attitude Index. Statistically significant difference in Concussion Knowledge Index	CK: + CA: =	None
16	Sullivan 2018	% n.a.; M_age_ = 14.47 ± 1.59	Younger adolescents	428	Ireland	Secondary school athletes	Pre-Post Experiment; 1–84	Theory-driven concussion education program based on Ajzen’s TPB; In-person; Digital	CK, CA, CS	The program has a significant positive effect on athletes’ knowledge, perceived behavioural control over concussion recognition and reporting. These results are maintained at 3 months follow-up. The program doesn't have a significant effect on athletes’ attitudes	CK: +	Theory of planned behaviour
17	Sullivan 2023	61.00%; M_age_:9–12	Younger adolescents	33	United States	Soccer	Pre-Post Experiment; 1	MPS VR app: semi-immersive VR concussion education app; Alone; Digital	CK, CA, CS	The mean concussion knowledge score significantly increased from pre- to postintervention. No significant changes in mean attitudes toward concussion reporting scores. Significant increase in mean concussion reporting intention scores from pre- to postintervention	CK: + CA: = C Reporting: =	None
18	Tallapragad 2022	50.67%; M_age_: n.a	Caregivers	600	United States	Football	Experiment; 1	Narrative source; In-person; Offline	other	Parents who read the narrative shared from the perspective of a high school football player, a parent, a coach, and a Center for Disease Control and Prevention researcher induced negative emotions, and those exposed to the narratives from the perspective of a high school football player and a parent also experienced a sense of identification and thereby reduced counterargument	Emotions: +	Narrative persuasion and Theory of choice
19	Wallace 2019	79.40%; M_age_: 16.02 ± 1.22	Older adolescents	102	United States	High school athletes	Pre-Post Experiment; 1	Concussion Bingo education program; In-person; Offline	CK	Student-athletes’ post-knowledge of SRC scores increased by 6 points or 8% (mainly knowledge related to some aspects e.g. some symptoms)	CK: +	Theory of planned behaviour
20	Warmath 2020	38.7%; M_age_ = 20.27 ± 2.25	Younger adolescents	465	United States	Competitive club sport athletes	RCT, 1	3 conditions; In-person; Mixed	other	Club sport athletes exposed to consequence based social marketing showed significantly higher levels of positive reporting beliefs and significantly lower levels of negative reporting beliefs than athletes exposed to traditional or revised symptom education	Positive beliefs: + Negative beliefs: -	Expectancy value theory
21	Wicklund 2021	68.4%; M_age_ = 15.1 ± 1.60	Younger adolescents	301	United States	Collision and contact sports	Pre-Post Experiment; 1	Vignette providing a definition of SRCs; In-person; Offline	CS	30% of adolescent athletes reported an increase in number of concussions after vignette overall no significant difference in reporting behaviour by age, sex, or sport type	Number of concussions: +	None
22	Zhou 2022	30.05%; A_Range_: >12	Caregivers	524 (Post intervention)	United States	Volleyball, basketball, soccer, football, or baseball	Pre-Post Experiment; 365	CDC HEADS UP handouts to parents; In-person; Offline	other	Percent of parents who talked to their child about concussion increased in the intervention group (CDC HEADS UP handouts)	CDC HEADS UP handouts: +	Theory of planned behaviour

Note: Younger adolescents aged 11–15 years; Older adolescents aged 16–20 years; CA, concussion awareness; CK, concussion knowledge; CS, concussion symptoms reporting behaviour; +, significant positive effect; −, significant negative effect; =, non-significant.

### Study Characteristics

The 22 studies included in this review were published between 2014 and 2023 (for a full reference list, see Supplementary Material). The distribution of publications is uniform over the past 10 years. Regarding geographical location, 17 studies were conducted in the US, four in Canada, and one in Ireland. Most studies (n = 15) used a single-arm design with pre-post assessment. Three studies used a randomized-controlled trial designs with pre-post assessment, and one study used a single-arm design with post-assessment only. Almost all studies evaluated short-term interventions ranging from one to 2 months. Only two interventions lasted for a longer time of approximately 1 year. Seven studies included a sample of up to 100 participants, ten between 101 and 500, and four more than 500. The mean sample size was 280. Most studies included participants from both sexes (n = 14), though most of the samples were composed of males (average % of males = 64.33). Sixteen studies analysed interventions targeted adolescent athletes aged 11 to 20, with only one reporting findings in young adults aged older than 18 on average. Caregivers of adolescent athletes were targeted in five of the included studies, and both athletes and their parents/caregivers in one study. Twelve studies reported on interventions focusing simultaneously on different sports, with one study including, among others, wrestling, which was retained as the study also focused on other team sports of interest in this review. Nine studies focused on one type of sport only. Included sports were, thus, American football, baseball, basketball, football, hockey, and wrestling. The most common intervention modality (n = 14) was a combination of digital and in-person elements (e.g., watching a film in the presence of others with an intervention from the moderator). In three studies, the intervention was only digital (e.g., through videos and mobile apps), while in five studies, it was exclusively held in person. Almost half of the studies (n = 10) based their intervention on CDC’s *Heads Up* campaign. Furthermore, twelve interventions were theory-based, with the majority applying the Theory of Planned Behaviour (n = 8). In each study, more than one outcome was analysed. Outcomes covered knowledge/awareness of SRCs (n = 12), attitudes (n = 6), symptom reporting behaviour (n = 8), and other outcomes such as SRC management, self-efficacy, emotions, and conversation with adolescents (n = 7). Table 1 summarizes the main study characteristics.

### Main Findings

#### Knowledge/Awareness

**FIGURE 2:**
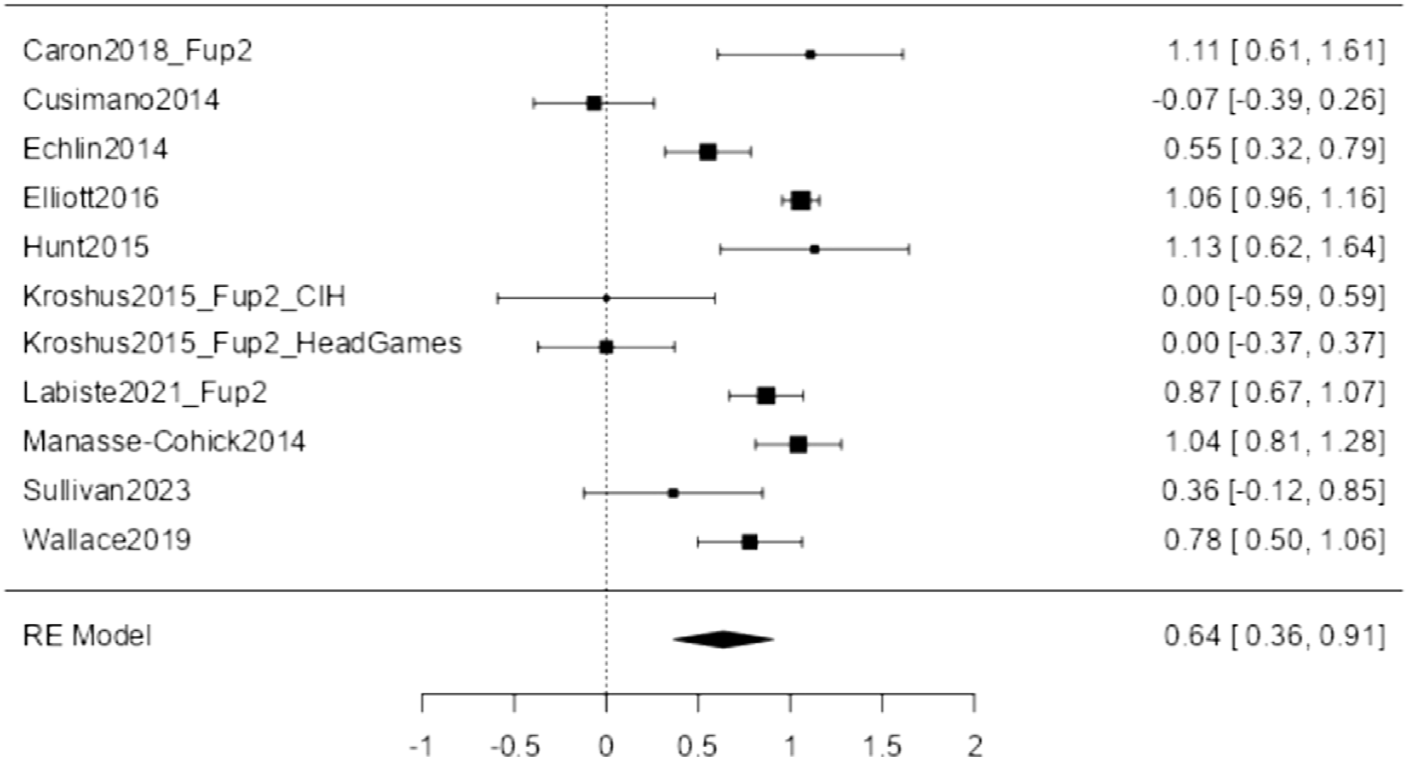
Forest plot of Sports-related Concussions Knowledge (Lugano, Switzerland, 2024).

Twelve studies addressed SRC awareness and knowledge, including how to prevent SRC, recognize concussion symptoms, and manage SRCs. Of these, ten studies provided the necessary data to be included in the meta-analysis comparing post-intervention to pre-intervention measures, with the article from Kroshus et al. [34] contributing two effect sizes as they compared two different interventions, thus *k* = 11. The observed standardized mean differences ranged from −0.067 to 1.132, with most of the estimates being positive (73%). The estimated average standardized mean difference based on the random-effects model was ^μ = 0.637 (95% CI: 0.364–0.910). Therefore, the average outcome differed significantly from zero (z = 4.569, *p* < 0.001). The analysis revealed heterogeneity (tau [2] = 0.1780, I^2^ = 91.062%). Hence, although the average outcome was estimated to be positive, in some studies, the true outcome may, in fact, be negative. Figure 2 shows the forest plot for sports-related conclusion (SRC) knowledge/awareness (*k* = 11).

Our moderator analysis showed no evidence of systematic differences due to sample composition (gender, age group), intervention modality, or time between pre- and post-assessment (see also Supplementary Table S7). Furthermore, neither the rank correlation nor Egger’s regression test showed any funnel plot asymmetry (p = 0.359 and p = 0.292, respectively), and we, therefore, have no evidence of publication bias (see also Supplementary Figure S1).

#### Attitudes

**FIGURE 3:**
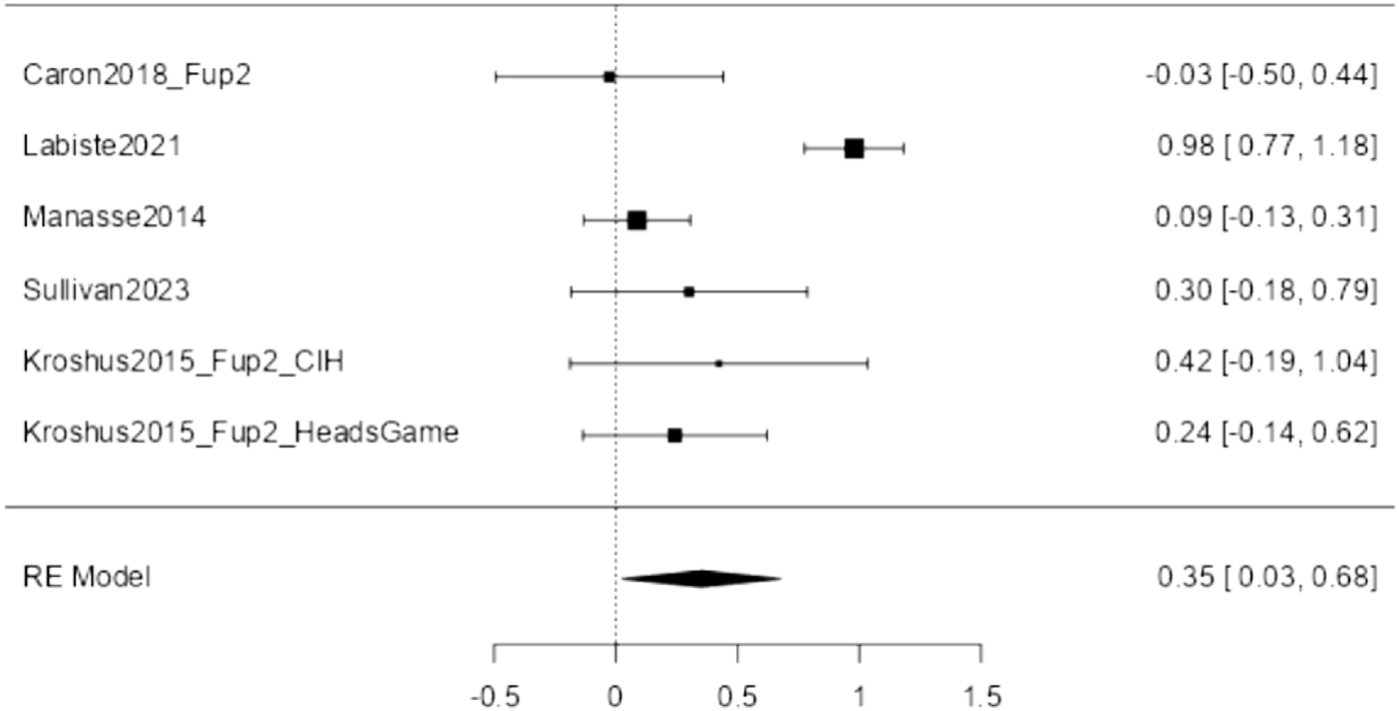
Forest plot of Sports-related Concussions Attitude (Lugano, Switzerland, 2024).

Six studies addressed the attitudes of adolescent athletes and/or their caregivers toward SRC, including risk perception and attitudes towards SRC prevention and early reporting. Of these, five studies provided the necessary data to be included in the meta-analysis comparing post-intervention to pre-intervention measures, with Kroshus et al. [34] contributing two effect sizes as they compared two different interventions, thus *k* = 6. The observed standardized mean differences ranged from −0.003 to 0.979, with most of the estimates being positive (83%). The estimated average standardized mean difference based on the random-effects model was ^μ = 0.352 (95% CI: 0.025–0.678). Therefore, the average outcome differed significantly from zero (z = 2.113, *p* = 0.035). The analysis revealed heterogeneity (tau [2] = 0.125, I^2^ = 81.50%). Figure 3 shows the forest plot for sports-related conclusion (SRC) attitudes (*k* = 6).

Moderator analysis showed no evidence of systematic differences due to gender, intervention modality, and time between pre- and post-assessment (see also Supplementary Table S11). In contrast, the intervention effect was larger in caregivers (^μ = 0.831 (95% CI: 0.572–1.090) compared to adolescents. Furthermore, neither the rank correlation nor Egger’s regression test indicated any funnel plot asymmetry (*p* = 0.719 and *p* = 0.477, respectively), again showing no evidence of publication bias (see also Supplementary Figure S2).

#### Symptoms Reporting Behaviour

**FIGURE 4:**
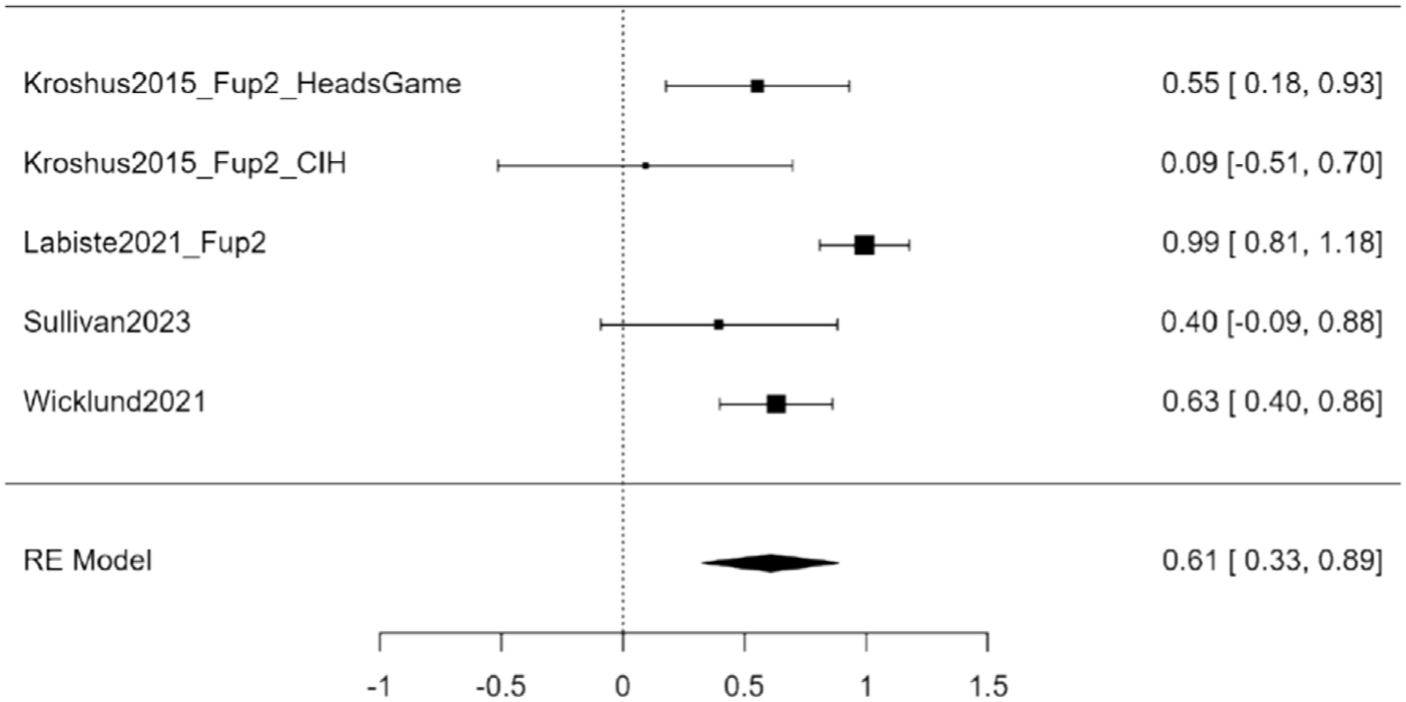
Forest plot of Sports-related Concussions Symptoms reporting behaviour (Lugano, Switzerland, 2024).

Eight studies focused on promoting the early reporting of symptoms by adolescent athletes or on emotions when reporting on symptoms. Of these, four studies provided the necessary data to be included in the meta-analysis comparing post-intervention to pre-intervention measures, with Kroshus et al. [34] contributing two effect sizes as they compared two different interventions, thus *k* = 5. The observed standardized mean differences ranged from 0.091 to 0.992, with all estimates being positive (100%). The estimated average standardized mean difference based on the random-effects model was ^μ = 0.530 (95% CI: 0.220–0.839). Therefore, the average outcome differed significantly from zero (z = 3.353, *p <* 0.001). The analysis revealed heterogeneity (tau [2] = 0.087, I^2^ = 78.05%). Figure 4 shows the forest plot for sports-related conclusion (SRC) symptoms reporting behaviour (*k* = 5).

Moderator analysis showed no evidence of systematic differences due to gender. In contrast, the intervention effect was larger in caregivers (^μ = 0.605 (95% CI: 0.352–0.858) compared to adolescents, where the outcome was soliciting SRC symptoms reporting by adolescent athletes (see also Supplementary Table S15). Furthermore, neither the rank correlation nor Egger’s regression test indicated any funnel plot asymmetry (*p* = 0.483 and *p* = 0.113, respectively), meaning that there was no evidence of publication bias (see also Supplementary Figure S3).

#### Other Outcomes

In addition to the above-mentioned outcomes, some studies also focused on other intervention outcomes, such as SRC management, self-efficacy, and communication among adolescent athletes and their parents. For example, in the study by Zhou et al. [54], the percentage of parents who talked to their child about concussion increased in the intervention group who received handouts with information developed as part of CDC’s *Heads Up* campaign. Another study showed that narratives from the perspectives of adolescent athletes, parents, coaches, or CDC researchers were more effective in promoting parents’ intention to talk to their children about SRC reporting than traditional, informative brochures.

### Study Quality

Regarding the quality and associated risk of bias, four articles had a poor quality (<50% of criteria fulfilled), 15 had a fairly good quality (50%–75% of criteria fulfilled), and only three had a very good quality (>75% of criteria fulfilled). Nearly all articles provided details on the intervention, the outcome(s), the applied statistical methodology, interpretation of results and limitations. Instead, the greatest amount of missing or insufficient information concerned sample recruitment and intervention implementation, including sequence generation, group allocation, implementation, and blinding.

## Discussion

Our systematic review and meta-analysis included 22 studies focusing on educating adolescent athletes and/or their caregivers about SRC prevention and early detection by increasing, among others, their awareness, knowledge, attitudes, and symptom reporting behaviour. The identified intervention studies differed from each other in terms of theoretical basis, methodology, and the studied outcomes. We derived three main findings that will be discussed in more detail. SRC communication and education interventions *(1) are more effective in improving awareness or knowledge, rather than improving attitudes, (2) tend to be short-term, and (3) their effects are independent of the target group, features, or outcome assessments*.

### Interventions Are More Effective in Improving Awareness and Knowledge Than Attitudes

Interventions designed to increase awareness and knowledge about concussions have demonstrated an overall positive standardized mean difference that was almost two times larger than for interventions designed to increase attitudes. However, it is important to emphasize that increased awareness and better knowledge are not sufficient in ensuring behaviour change [41, 55, 56]. Merely increasing awareness of risks or know-how to protect oneself from mTBI in sports contexts may not be followed by concrete actions. The transformation of knowledge into behaviour is a complex process influenced by multiple individual and contextual factors, including attitude change. Attitudes are important as they influence individuals’ decisions and behaviors. In fact, according to established health behaviour theories such as the Theory of Planned Behaviour or the Health Belief Model, better awareness, and knowledge of SRCs contribute to an adequate risk assessment and more positive beliefs and attitudes towards SRC prevention, which are necessary to guide actual SRC prevention behaviour. Nonetheless, theory and reality may not always match as it was found that, despite risk awareness and knowledge, adolescents may maintain attitudes that minimise the importance of preventive measures or encourage risky behaviour [57].

Our review is not the only one identifying different results for knowledge gain and attitudes change. Similar results were found in a systematic review by Conaghan and colleagues [32]: they analysed the effectiveness of concussion prevention programs on knowledge and attitudes in different age groups engaged in sports. The authors found that, in almost all studies, there was an increase in knowledge (17 out of 20), but less frequently a change in attitudes (13 out of 20). Our results are compelling considering that adolescent athletes are particularly pressured by their environment and social norms [58]. Thus, awareness, knowledge, and positive attitudes towards SRC prevention are fundamental to resist peer pressure to refrain from protective behaviours, such as wearing a helmet (“*It’s so uncool!*”), or to cope with negative evaluation from teammates when reporting concussion-related symptoms (“*Don’t be a wimp!*”). A need to overcome the potentially negative impact of peer pressure and evaluation through solid knowledge and attitudes has also been observed in other health contexts, such as vaccination or the prevention of sexually transmitted diseases [44, 45]. Therefore, bridging the gap between knowledge gain and attitudes change requires an integrated approach. This approach should include communication and education efforts that not only inform about the symptoms and seriousness of SRCs, and how to take precautionary measures, but also address the psychological, social, and systemic barriers that may hinder the adoption of protective and preventive behaviours [33, 47]. To note, in our review, we found a positive and significant pooled effect of interventions on athletes’ SRC symptoms reporting behaviour, which is crucial given that research has evidenced under-reporting of SRC by adolescent athletes [59]. Future studies should specifically test the effectiveness of integrative, theory-driven interventions in translating SRC-related knowledge into sustained behavioural change. Despite the positive and statistically significant pooled effect sizes observed in our meta-analyses, the high heterogeneity across studies indicates that the underlying true effects likely vary substantially between interventions. This limits the precision and generalizability of the average estimates. Although we applied a random-effects model and tested potential moderators, these sources of variation were only partially explained. Therefore, the pooled outcomes should be interpreted with caution, as they represent aggregated effects from diverse populations, interventions, and measurement tools.

### Interventions and Their Effects Tend to Be Short-Term

Almost all the studies in our review focused on short-term interventions ranging from one to 2 months. Only two interventions lasted for a longer time of approximately 1 year. However, evidence exists that repeated retrieval of information significantly improves retention due to the processes of memory consolidation and reconsolidation [60], and this takes time. It should also be noted that SRC among adolescent athletes are particularly complex and influenced by multiple variables. Furthermore, athletes may receive contrasting information, make their own experiences, or be differently influenced or supported by their social context (including caregivers and coaches). Therefore, interventions must take place over a longer period to attract the target groups to pay attention, understand, consolidate, and store information in one’s long-term memory or, simply put, acquire knowledge [61]. Likewise, time is needed to change attitudes and, eventually, behaviour, as strongly held attitudes are rather stable and resistant to education interventions and persuasion efforts [62].

Linked to the duration of SRC prevention interventions is also the time lag between the intervention and the assessment of the expected outcomes. The time lag in the included intervention studies ranged from 1 to 365 days, and seven studies conducted multiple follow-up assessments at different timepoints. Despite short-term effects in improving concussion awareness/knowledge, attitude and symptoms reporting behaviour, these tend to fade out over time, meaning that current SRC interventions may not be sustainable in the long term [63]. In fact, studies have shown that people can experience habit slips [41, 62], which requires repeated reminders for a SRC prevention intervention to be effective and the “right” timing of assessment to observe its effectiveness. To enhance long-term effectiveness, future programs should incorporate booster sessions, periodic refreshers, or integration into routine sports education curricula, enabling continuous reinforcement of key messages. Embedding SRC education into regular team meetings or training cycles could help maintain knowledge and support gradual attitude change over time.

### Intervention Effects Are Independent of the Sports Context, Intervention Features or Target Group

The last finding of this review is that the effectiveness of SRC interventions aimed at adolescent athletes and their caregivers does not seem to depend on which sport is considered, the features of the intervention, the gender, or the age of adolescents. One exception are interventions aimed at caregivers, where stronger effects were found for attitudes change and soliciting SRC symptoms reporting behaviour by adolescent athletes. This may be because caregivers play a central role in health-related decision-making and are often the ones enforcing or encouraging protective behaviours [64]. Their involvement adds a layer of supervision and advocacy that complements educational efforts targeting the athletes themselves. Moreover, caregivers may be more receptive to health messages and can act as consistent reinforcers of safe behaviour over time, especially when adolescent motivation fluctuates. Across all the interventions included in this review, whether they used classic lectures, a Bingo game, videos, or interviews with parents, no noteworthy differences could be found based on specific contextual or intervention characteristics. One reason might be that there is still a lack of a solid theoretical basis guiding the development and implementation of SRC prevention campaigns. Twelve out of 22 studies in this review reported that their intervention was based on theory, with most studies applying behaviour change theory, such as the Theory of Planned Behaviour. Only two used message design or perception and elaboration theories such as the Elaboration Likelihood Model, that help identify when and which message features should be used to capture people’s attention and facilitate message processing. This requires a paradigm shift where public health organisations, most likely commissioning and developing communication and education interventions, collaborate and learn from health communication experts with strong backgrounds in theory-based health campaign development. Nonetheless, more rigorous, comparative research is needed to determine whether specific delivery formats (e.g., digital vs. in-person) may be more effective in certain contexts or populations.

### Limitations and Future Directions

There are some limitations pertaining to the articles included in this review. First, almost all the interventions were carried out in the US and Canada, which may be because the high-risk sports considered in this review are more popular in these countries. However, this limits the generalizability of the findings to a subset of WEIRD (i.e., Western, Educated, Industrialized, Rich, and Democratic) populations [65], which emphasizes the need for further studies in other European countries and underrepresented regions such as Asia, Africa, and Latin America. Second, the sample size of 7 out of 22 studies was generally small, i.e., including less than 100 participants. Third, almost all studies limited their interventions to one to 2 months. Small samples and short durations are frequently reported in intervention studies [66]. Yet, they limit the detection of small but significant effects due to being underpowered and incapable of detecting dose-response mechanisms that evolve over time. Fourth, not all included studies reported the necessary data to be included in the meta-analysis. Fifth, the measured outcomes varied considerably in concept, format, and validity, which reduced the number of studies to be included in the meta-analytic part to few, comparable effect sizes. Lastly, the reporting quality varied considerably among the included articles, with only three providing sufficient detail and using state-of-the-art methodologies to be considered of very good quality.

Finally, three limitations that concern our search strategy should be acknowledged. We included only articles published in English and in peer-reviewed journals. Thus, we may have missed studies of high quality and informative value published in other languages and/or in the grey literature. Yet, publishing in English-speaking peer-reviewed journals is the state-of-the-art in scientific research and we considered it an additional quality marker of the included studies in our review. Furthermore, we limited our search to title and abstract to increase precision and reduce the number of irrelevant records, particularly given the interdisciplinary nature of SRC-related terminology. While this may have reduced sensitivity, it allowed for more targeted screening [67]. Additionally, only one reviewer (R1) conducted the full-text screening and data extraction. Although a second reviewer (R2) cross-checked three randomly selected articles, the reliance on a single primary reviewer is a constraint that may introduce bias and thus affect objectivity and consistency of the review process.

Considering these limitations, future research should prioritise conducting rigorous and adequately powered randomised controlled trials (RCTs) with larger sample sizes and extended intervention periods. These studies should use standardised outcome measures, such as the Rosenbaum Concussion Knowledge and Attitudes Survey (RoCKAS) [68], to allow for better comparability between studies. In addition, expanding the research to underrepresented regions, including the Global South, will improve the global applicability of findings. Without such diversity, any recommendations remain limited in their cultural, contextual, and sport-specific relevance. These steps are essential to generalise the effectiveness of interventions across diverse populations. Moreover, greater collaboration between health communication experts, public health organisations, and coaches could facilitate the development of interventions based on sound theoretical models that can foster the behavioural change necessary for SRC prevention and early detection in adolescent athletes. Finally, cultural differences in how SRCs are perceived, reported, and managed may influence both intervention effectiveness and outcome reporting, underscoring the need for context-sensitive approaches in future research.

### Conclusions

This systematic literature review and meta-analysis summarized evidence on the effectiveness of communication and education interventions targeting adolescent athletes and their caregivers with the aim to prevent severe outcomes of SRCs in adolescent athletes. We found empirical support for improved awareness and knowledge of SRC, SRC symptoms reporting behaviour and SRC-related attitudes. Furthermore, interventions tended to be short-term, and effectiveness generally did not depend on the characteristics of the target populations, intervention implementation, or outcome measure. More interventions and their evaluation are needed to corroborate these findings.
