# Supplementary material for: Mitigating Sports-Related Concussions in Adolescent Athletes: A Systematic Review and Meta-Analysis of Communication and Education Interventions
Source: Public Health Rev. 2025 May 16;46:1608153. doi: 10.3389/phrs.2025.1608153 (PMC12122304; doi:10.3389/phrs.2025.1608153)
Supplement: Supplementary file 1 [file DataSheet1.docx]

**Supplement**

**Mitigating Sports-Related Mild Traumatic Brain Injuries:**

**A Systematic Review and Meta-Analysis of Communication Interventions**

[Supplement Table 1: Keywords for search strategy 2](#_Toc167718895)

[Supplement Table 2: Quality assessment checklist 3](#_Toc167718896)

[Supplement Table 3: Quality assessment of included articles (n=22) 5](#_Toc167718897)

[Supplement: Additional meta-analysis results for knowledge/awareness 6](#_Toc167718898)

[Supplement: Additional meta-analysis results for attitudes 8](#_Toc167718899)

[Supplement: Additional meta-analysis results for symptoms reporting behaviour 10](#_Toc167718900)

[Supplement: References list of included article (n=22) 12](#_Toc167718901)

# Supplement Table 1: Keywords for search strategy

| Population | young OR youth OR child* OR adolescen* OR teen* OR parent* OR caregiver* OR mother OR father OR carer* |
| --- | --- |
|  | AND |
| Intervention | campaign OR educat* OR teach* OR instruct* OR communication* OR intervent* OR inform* OR initiative* |
|  | AND |
| Comparison | - |
|  | AND |
| Outcome | - |
|  | AND |
| Setting 1 | (sport* OR activit* OR athlet* OR exercis* OR train* OR elite OR agonis* OR competition* OR competitive* |
|  | AND |
| Setting 2 | concussion* OR (head AND trauma*) OR (brain AND injur*) OR (mTBI) OR (mild AND traumatic AND brain AND injury |
|  | NOT |
| Exclusion | (brain AND imaging) OR (electroencephalogr*) OR (ecg) OR (axon*) OR (abus*) OR (hemophilia) OR (spin*) OR (distortion) OR (home) OR (house) |

*Note: Searched in title and abstract only*

# Supplement Table 2: Quality assessment checklist

| 1. Title and abstract | 1a Identification as a randomised trial in the title |
| --- | --- |
|  | 1b Structured summary of trial design, methods, results, and conclusions (for specific guidance see CONSORT for abstracts) |
| 2. Introduction | 2a Scientific background and explanation of rationale |
|  | 2b Specific objectives or hypotheses |
| 3. Methods | Description of trial design (such as parallel, factorial) including allocation ratio |
| 4. Participants | 4a Eligibility criteria for participants |
|  | 4b Settings and locations where the data were collected |
| 5. Interventions | The interventions for each group with sufficient details to allow replication, including how and when they were actually administered |
| 6. Outcomes | Completely defined pre-specified primary and secondary outcome measures, including how and when they were assessed |
| 7. Sample size | 7a How sample size was determined |
|  | 7b When applicable, explanation of any interim analyses and stopping guidelines |
| 8. Sequence generation | 8a Method used to generate the random allocation sequence |
|  | 8b Type of randomisation; details of any restriction (such as blocking and block size) |
| 9. Allocation concealment mechanism | Mechanism used to implement the random allocation sequence (such as sequentially numbered containers), describing any steps taken to conceal the sequence until interventions were assigned |
| 10. Implementation | Who generated the random allocation sequence, who enrolled participants, and who assigned participants to interventions |
| 11. Blinding | 11a If done, who was blinded after assignment to interventions (for example, participants, care providers, those assessing outcomes) and how |
|  | 11b If relevant, description of the similarity of interventions |
| 12. Statistical methods | 12a Statistical methods used to compare groups for primary and secondary outcomes |
|  | 12b Methods for additional analyses, such as subgroup analyses and adjusted analyses |
| 13. Participant flow (a diagram is strongly recommended) | 13a For each group, the numbers of participants who were randomly assigned, received intended treatment, and were analysed for the primary outcome |
|  | 13b For each group, losses and exclusions after randomisation, together with reasons |
| 14. Recruitment | Dates defining the periods of recruitment and follow-up |
| 15. Baseline data | A table showing baseline demographic and clinical characteristics for each group |
| 16. Numbers analyzed | For each group, number of participants (denominator) included in each analysis and whether the analysis was by original assigned groups |
| 17. Outcomes and estimation | 17a For each primary and secondary outcome, results for each group, and the estimated effect size and its precision (such as 95% confidence interval) |
|  | 17b For binary outcomes, presentation of both absolute and relative effect sizes is recommended |
| 18. Ancillary analysis | Results of any other analyses performed, including subgroup analyses and adjusted analyses, distinguishing pre-specified from exploratory |
| 19. Harms | All important harms or unintended effects in each group (for specific guidance see CONSORT for harms) |
| 20. Limitations | Trial limitations, addressing sources of potential bias, imprecision, and, if relevant, multiplicity of analyses |
| 21. Generalisability | Generalisability (external validity, applicability) of the trial findings |
| 22. Interpretation | Interpretation consistent with results, balancing benefits and harms, and considering other relevant evidence |
| 23. Registration | Registration number and name of trial registry |
| 24. Protocol | Where the full trial protocol can be accessed, if available |
| 25. Funding | Sources of funding and other support (such as supply of drugs), role of funders |

*Note: Adapted from CONSORT checklist for RCTs*

| **Nr** | **FirstAuthor Year** | **1a** | **1b** | **2a** | **2b** | **3** | **4a** | **4b** | **5** | **6** | **7a** | **7b** | **8a** | **8b** | **9** | **10** | **11a** | **11b** | **12a** | **12b** | **13a** | **13b** | **14** | **15** | **16** | **17a** | **17b** | **18** | **19** | **20** | **21** | **22** | **23** | **24** | **25** | **Sum** | **Max** | **%Scoring** |
| --- | --- | --- | --- | --- | --- | --- | --- | --- | --- | --- | --- | --- | --- | --- | --- | --- | --- | --- | --- | --- | --- | --- | --- | --- | --- | --- | --- | --- | --- | --- | --- | --- | --- | --- | --- | --- | --- | --- |
| 1 | Caron2018 | 1 | 1 | 1 | 1 | 0.5 | 0 | 1 | 0 | 1 | 0 | 0 | 0 | 0 | 0 | 0 | 0 | 0 | 1 | 1 | 0 | 0 | 0 | 0 | 1 | 1 | 1 | 1 | 0 | 1 | 1 | 1 | 0 | 0 | 0 | 16.5 | 31 | 53.2 |
| 2 | Cranmer2021 | 0 | 1 | 1 | 1 | 0.5 | 0 | 0 | 1 | 1 | 0 | 0 | 0 | 0 | 0 | 0 | 0 | 0 | 1 | 0 | 0 | 0 | 0 | 0 | 0 | 1 | 1 | 0 | 0 | 1 | 0.5 | 1 | 0 | 0 | 0 | 12 | 31 | 38.7 |
| 3 | Cusimano2014 | 1 | 1 | 1 | 1 | 1 | 0 | 0 | 1 | 1 | 0 | 0 | 0 | 0 | 0 | 0 | 0 | 0 | 1 | 1 | 1 | 1 | 0 | 0 | 1 | 1 | 1 | 1 | 0 | 1 | 0 | 1 | 0 | 0 | 1 | 19 | 31 | 61.3 |
| 4 | Eagles2016 | 0 | 1 | 1 | 1 | 1 | 0 | 1 | 0.5 | 1 | 0 | 0 | n.a. | n.a. | n.a. | n.a. | n.a. | 0 | 1 | 0 | n.a. | n.a. | 0 | 1 | 0.5 | 1 | 0 | 0 | 0 | 1 | 0.5 | 1 | 0 | 0 | 0 | 13.5 | 24 | 56.3 |
| 5 | Echlin2014 | 0 | 0.5 | 1 | 0.5 | 1 | 0.5 | 1 | 1 | 1 | 0 | 0 | n.a. | n.a. | n.a. | n.a. | n.a. | 0 | 1 | 1 | n.a. | n.a. | 0 | 0 | 1 | 1 | 1 | 0 | 0 | 1 | 0.5 | 1 | 0 | 0 | 1 | 16 | 24 | 66.7 |
| 6 | Elliott2016 | 0 | 1 | 1 | 1 | 1 | 0 | 0..5 | 1 | 1 | 0 | 0 | n.a. | n.a. | n.a. | n.a. | n.a. | 0 | 1 | 1 | n.a. | n.a. | 0 | 1 | 1 | 1 | 1 | 0 | 0 | 1 | 1 | 1 | 0 | 0 | 1 | 17 | 24 | 70.8 |
| 7 | Glang2015 | 0 | 1 | 1 | 1 | 1 | 1 | 0.5 | 1 | 1 | 1 | 0 | 0 | 1 | 0.5 | 0.5 | 0 | 0 | 1 | 1 | 1 | 0 | 0 | 1 | 1 | 1 | 1 | 1 | 0 | 1 | 1 | 1 | 0 | 1 | 1 | 23.5 | 31 | 75.8 |
| 8 | Hunt2015 | 0 | 1 | 1 | 1 | 1 | 1 | 0 | 1 | 1 | 0 | 0 | n.a. | n.a. | n.a. | n.a. | n.a. | 0 | 1 | 1 | n.a. | n.a. | 0 | 1 | 0 | 0 | 1 | 1 | 0 | 1 | 1 | 1 | 0 | 0 | 0 | 16 | 24 | 66.7 |
| 9 | Kantorski2020 | 0 | 1 | 1 | 0.5 | 1 | 0 | 0 | 1 | 0.5 | 0 | 0 | n.a. | n.a. | n.a. | n.a. | n.a. | 0 | 1 | 0 | n.a. | n.a. | 0 | 1 | 0 | 0 | 0 | 0 | 0 | 1 | 0.5 | 1 | 0 | 1 | 1 | 12.5 | 24 | 52.1 |
| 10 | Kroshus2015 | 1 | 1 | 1 | 1 | 1 | 1 | 0 | 1 | 1 | 0 | 0 | 0 | 1 | 0 | 0 | 0 | 0 | 1 | 1 | 1 | 0 | 1 | 0 | 1 | 1 | 1 | 1 | 0 | 1 | 1 | 1 | 0 | 0 | 1 | 22 | 31 | 71.0 |
| 11 | Kroshus2023 | 1 | 1 | 1 | 1 | 1 | 0.5 | 0 | 1 | 1 | 0 | 0 | 0 | 1 | 0 | 0 | 0 | 0 | 1 | 1 | 1 | 1 | 1 | 1 | 1 | 0 | 0 | 0 | 0 | 1 | 1 | 1 | 1 | 1 | 1 | 22.5 | 31 | 72.6 |
| 12 | Kurowski2015 | 0 | 1 | 1 | 1 | 1 | 0 | 0.5 | 0.5 | 1 | 0 | 0 | n.a. | n.a. | n.a. | n.a. | n.a. | 0 | 1 | 1 | n.a. | n.a. | 0 | 1 | 1 | 0 | 0 | 0 | 0 | 1 | 0.5 | 1 | 0 | 0 | 1 | 14.5 | 24 | 60.4 |
| 13 | Labiste2021 | 0 | 1 | 1 | 1 | 1 | 1 | 0 | 1 | 1 | 0.5 | 0 | n.a. | n.a. | n.a. | n.a. | n.a. | 0 | 1 | 1 | n.a. | n.a. | 0 | 0 | 1 | 1 | 1 | 1 | 0 | 1 | 0 | 1 | 0 | 0 | 1 | 17 | 24 | 70.8 |
| 14 | Macdonald2016 | 0 | 1 | 1 | 0.5 | 1 | 1 | 0 | 1 | 1 | 0 | 0 | n.a. | n.a. | n.a. | n.a. | n.a. | 0 | 1 | 0 | n.a. | n.a. | 0 | 0 | 1 | 0 | 0 | 0 | 0 | 0 | 0.5 | 1 | 0 | 0 | 0 | 11 | 24 | 45.8 |
| 15 | Manasse-Cohick2014 | 0 | 0.5 | 1 | 1 | 1 | 0 | 1 | 1 | 1 | 0 | 0 | n.a. | n.a. | n.a. | n.a. | n.a. | 0 | 1 | 0 | n.a. | n.a. | 0 | 0 | 1 | 0 | 0 | 0 | 0 | 1 | 0 | 1 | 0 | 0 | 1 | 12.5 | 24 | 52.1 |
| 16 | Sullivan2018 | 0 | 1 | 1 | 1 | 1 | 1 | 1 | 1 | 1 | 1 | 0 | n.a. | n.a. | n.a. | n.a. | n.a. | 0 | 1 | 1 | n.a. | n.a. | 1 | 1 | 1 | 1 | 1 | 1 | 0 | 1 | 1 | 1 | 0 | 1 | 1 | 22 | 24 | 91.7 |
| 17 | Sullivan2023 | 0 | 1 | 1 | 1 | 1 | 1 | 0 | 1 | 1 | 0 | 0 | n.a. | n.a. | n.a. | n.a. | n.a. | 0 | 1 | 1 | n.a. | n.a. | 1 | 0 | 0 | 1 | 1 | 1 | 0 | 1 | 1 | 1 | 0 | 0 | 0 | 17 | 24 | 70.8 |
| 18 | Tallapragad2022 | 0 | 1 | 1 | 1 | 1 | 0 | 0 | 1 | 1 | 0 | 0 | 0 | 0.5 | 0 | 0 | 0 | 0 | 1 | 1 | 1 | 0 | 0 | 0.5 | 1 | 1 | 1 | 1 | 0 | 1 | 1 | 1 | 0 | 0 | 1 | 19 | 31 | 61.3 |
| 19 | Wallace2019 | 0 | 1 | 1 | 1 | 1 | 0.5 | 0.5 | 1 | 1 | 0 | 0 | 0 | 0 | 0 | 0 | 0 | 0 | 1 | 1 | 0 | 0 | 0 | 1 | 1 | 1 | 1 | 1 | 0 | 1 | 1 | 1 | 0 | 0 | 1 | 19 | 31 | 61.3 |
| 20 | Warmath2020 | 0 | 1 | 1 | 1 | 1 | 0 | 0 | 1 | 1 | 1 | 0 | 1 | 1 | 1 | 1 | 1 | 0 | 1 | 1 | 1 | 0 | 1 | 1 | 1 | 1 | 1 | 1 | 0 | 1 | 1 | 1 | 0 | 0 | 1 | 25 | 31 | 80.6 |
| 21 | Wicklund2021 | 0 | 1 | 1 | 1 | 1 | 0 | 0 | 0.2 | 0 | 0 | 0 | n.a. | n.a. | n.a. | n.a. | n.a. | 0 | 1 | 1 | n.a. | n.a. | 0 | 1 | 1 | 0 | 0 | 0 | 0 | 1 | 0 | 1 | 0 | 0 | 0 | 11.2 | 24 | 46.7 |
| 22 | Zhou2022 | 0 | 1 | 1 | 1 | 1 | 0.5 | 0.5 | 1 | 1 | 0 | 0 | 1 | 1 | 0 | 0 | 0 | 0 | 1 | 1 | 1 | 0 | 0 | 1 | 1 | 1 | 1 | 0 | 0 | 1 | 1 | 1 | 0 | 0 | 1 | 20 | 31 | 64.5 |

# Supplement Table 3: Quality assessment of included articles (n=22)

# Supplement: Additional meta-analysis results for knowledge/awareness

**Meta-analysis for knowledge/awareness including single-arm and RCT studies (intervention group) with pre-post-assessment (*k*=11)**

| Supplement Table 5 : Random-Effects Model (*k* = 11) | | | | | | | | | | | | | |
| --- | --- | --- | --- | --- | --- | --- | --- | --- | --- | --- | --- | --- | --- |
|  | | **Estimate** | | **se** | | **Z** | | **p** | | **CI Lower Bound** | | **CI Upper Bound** | |
| Intercept |  | 0.637 |  | 0.139 |  | 4.57 |  | < .001 |  | 0.364 |  | 0.910 |  |
| Note. Tau² Estimator: Restricted Maximum-Likelihood | | | | | | | | | | | | | |

| Supplement Table 6 : Heterogeneity Statistics | | | | | | | | | | | | | | | |
| --- | --- | --- | --- | --- | --- | --- | --- | --- | --- | --- | --- | --- | --- | --- | --- |
| **Tau** | | **Tau²** | | **I²** | | **H²** | | **R²** | | **df** | | **Q** | | **p** | |
| 0.424 |  | 0.1795 (SE= 0.0951) |  | 91.06% |  | 11.188 |  |  |  | 10.000 |  | 89.655 |  | < .001 |  |

Supplement Table 7 : Summary of Moderator Analysis: Mixed-Effects Model (*k* = 11)

|  | | **Estimate** | | **se** | | **Z** | | **p** | | **CI Lower Bound** | | **CI Upper Bound** | |
| --- | --- | --- | --- | --- | --- | --- | --- | --- | --- | --- | --- | --- | --- |
| Intercept |  | 1.317 |  | 0.550 |  | 2.40 |  | 0.017 |  | 0.240 |  | 2.394 |  |
| Gender (%male)^1^ |  | -0.919 |  | 0.719 |  | -1.28 |  | 0.201 |  | -2.329 |  | 0.491 |  |
| Intercept |  | 0.7064 |  | 0.455 |  | 1.553 |  | 0.120 |  | -0.185 |  | 1.598 |  |
| Age category^2^ |  | -0.0487 |  | 0.295 |  | -0.165 |  | 0.869 |  | -0.627 |  | 0.529 |  |
| Intercept |  | 0.350 |  | 0.552 |  | 0.634 |  | 0.526 |  | -0.731 |  | 1.431 |  |
| Age category^3^ |  | 0.259 |  | 0.483 |  | 0.537 |  | 0.591 |  | -0.687 |  | 1.206 |  |
| Intercept |  | 0.430 |  | 0.472 |  | 0.911 |  | 0.362 |  | -0.495 |  | 1.354 |  |
| Intervention modality |  | 0.133 |  | 0.291 |  | 0.459 |  | 0.646 |  | -0.436 |  | 0.703 |  |
| Intercept |  | 0.8451 |  | 0.2706 |  | 3.123 |  | 0.002 |  | 0.315 |  | 1.375 |  |
| Time between pre- and  post-assessment |  | -0  0698 |  | 0.0773 |  | -0.903 |  | 0.366 |  | -0.221 |  | 0.082 |  |
| Note. Tau² Estimator: Restricted Maximum-Likelihood ; ^1^*k* = 8 since gender was not always reported in the included studies; ^2^ Reference group: 1 = young adolescents; ^3^Reference group: 1 = caregivers | | | | | | | | | | | | | |

| Supplement Table 8 : Publication Bias Assessment | | | | | |
| --- | --- | --- | --- | --- | --- |
| **Test Name** | | **value** | | **p** | |
| Fail-Safe N |  | 1210.000 |  | < .001 |  |
| Begg and Mazumdar Rank Correlation |  | -0.236 |  | 0.359 |  |
| Egger's Regression |  | -1.054 |  | 0.292 |  |
| Trim and Fill Number of Studies |  | 0.000 |  | . |  |
| Note. Fail-safe N Calculation Using the Rosenthal Approach | | | | | |

Supplement Figure 1 : Funnel Plot


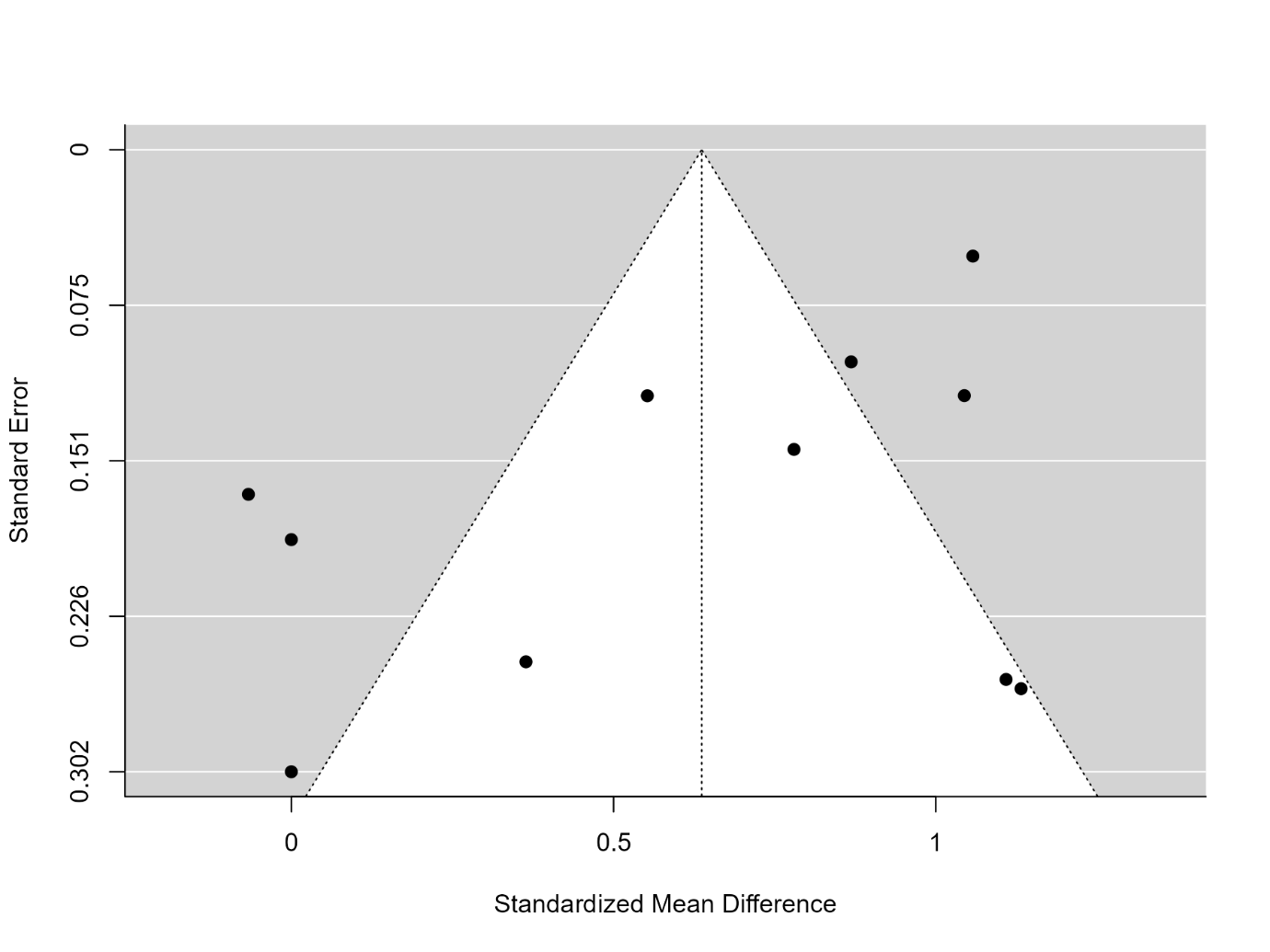


# Supplement: Additional meta-analysis results for attitudes

**Meta-analysis for attitude including single-arm and RCT studies (intervention group) with pre-post-assessment (*k*=6)**

| Supplement Table 9 : Random-Effects Model (*k* = 6) | | | | | | | | | | | | | |
| --- | --- | --- | --- | --- | --- | --- | --- | --- | --- | --- | --- | --- | --- |
|  | | **Estimate** | | **se** | | **Z** | | **p** | | **CI Lower Bound** | | **CI Upper Bound** | |
| Intercept |  | 0.352 |  | 0.166 |  | 2.11 |  | 0.035 |  | 0.025 |  | 0.678 |  |
| Note. Tau² Estimator: Restricted Maximum-Likelihood | | | | | | | | | | | | | |

| Supplement Table 10 : Heterogeneity Statistics | | | | | | | | | | | | | | | |
| --- | --- | --- | --- | --- | --- | --- | --- | --- | --- | --- | --- | --- | --- | --- | --- |
| **Tau** | | **Tau²** | | **I²** | | **H²** | | **R²** | | **df** | | **Q** | | **p** | |
| 0.354 |  | 0.1251 (SE= 0.1044) |  | 81.50% |  | 5.407 |  | . |  | 5.000 |  | 41.844 |  | < .001 |  |

Supplement Table 11 : Summary of Moderator Analysis: Mixed-Effects Model (*k* = 6)

|  | | **Estimate** | | **se** | | **Z** | | **p** | | **CI Lower Bound** | | **CI Upper Bound** | |
| --- | --- | --- | --- | --- | --- | --- | --- | --- | --- | --- | --- | --- | --- |
| Intercept |  | 0.474 |  | 0.669 |  | 0.709 |  | 0.479 |  | -0.837 |  | 1.786 |  |
| Gender (%male)^1^ |  | -0.284 |  | 0.724 |  | -0.392 |  | 0.695 |  | -1.702 |  | 1.134 |  |
| Intercept |  | 0.4134 |  | 0.606 |  | 0.681 |  | 0.496 |  | -0.775 |  | 1.602 |  |
| Age category^2^ |  | -0.056 |  | 0.503 |  | -0.111 |  | 0.911 |  | -1.041 |  | 0.930 |  |
| Intercept |  | -0.684 |  | 0.193 |  | -3.54 |  | <.001 |  | -1.063 |  | -0.305 |  |
| Age category^3^ |  | 0.831 |  | 0.132 |  | 6.29 |  | <.001 |  | 0.572 |  | 1.090 |  |
| Intercept |  | 0.742 |  | 0.523 |  | 1.417 |  | 0.156 |  | -0.284 |  | 1.768 |  |
| Intervention modality |  | -0.270 |  | 0.342 |  | -0.790 |  | 0.429 |  | -0.940 |  | 0.400 |  |
| Intercept |  | -0.127 |  | 0.274 |  | -0.463 |  | 0.643 |  | -0.663 |  | 0.410 |  |
| Time between pre- and  post-assessment |  | 0.220 |  | 0.108 |  | 2.034 |  | 0.042 |  | 0.008 |  | 0.432 |  |
| Note. Tau² Estimator: Restricted Maximum-Likelihood; ^1^*k* = 4 since gender was not always reported in the included studies; ^2^ Reference group: 1 = young adolescents; ^3^Reference group: 1 = caregivers | | | | | | | | | | | | | |

| Supplement Table 12 : Publication Bias Assessment | | | | | |
| --- | --- | --- | --- | --- | --- |
| **Test Name** | | **value** | | **p** | |
| Fail-Safe N |  | 66.000 |  | < .001 |  |
| Begg and Mazumdar Rank Correlation |  | 0.200 |  | 0.719 |  |
| Egger's Regression |  | -0.711 |  | 0.477 |  |
| Trim and Fill Number of Studies |  | 1.000 |  | . |  |
| Note. Fail-safe N Calculation Using the Rosenthal Approach | | | | | |

Supplement Figure 2 : Funnel Plot


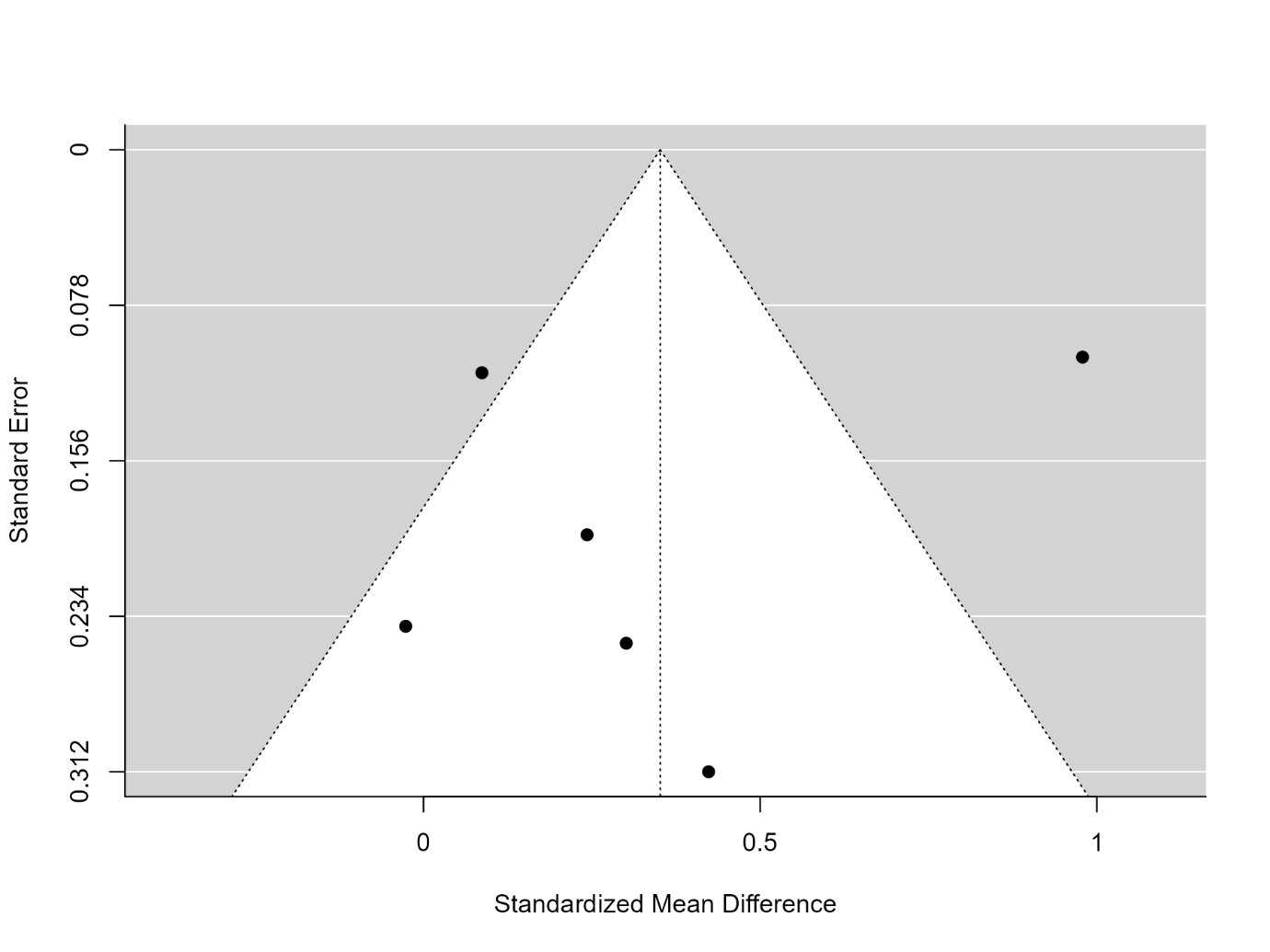


# Supplement: Additional meta-analysis results for symptoms reporting behaviour

**Meta-analysis for symptoms reporting behaviour including single-arm and RCT studies (intervention group) with pre-post-assessment (*k* = 5)**

| Supplement Table 13 : Random-Effects Model (*k* = 5) | | | | | | | | | | | | | |
| --- | --- | --- | --- | --- | --- | --- | --- | --- | --- | --- | --- | --- | --- |
|  | | **Estimate** | | **se** | | **Z** | | **p** | | **CI Lower Bound** | | **CI Upper Bound** | |
| Intercept |  | 0.530 |  | 0.158 |  | 3.35 |  | <.001 |  | 0.220 |  | 0.839 |  |
| Note. Tau² Estimator: Restricted Maximum-Likelihood | | | | | | | | | | | | | |

| Supplement Table 14 : Heterogeneity Statistics | | | | | | | | | | | | | | | |
| --- | --- | --- | --- | --- | --- | --- | --- | --- | --- | --- | --- | --- | --- | --- | --- |
| **Tau** | | **Tau²** | | **I²** | | **H²** | | **R²** | | **df** | | **Q** | | **p** | |
| 0.298 |  | 0.0886(SE=0.0876) |  | 78.05% |  | 4.556 |  | . |  | 4.000 |  | 23.646 |  | <.001 |  |

Supplement Table 15 : Summary of Moderator Analysis: Mixed-Effects Model (*k* = 5)

|  | | **Estimate** | | **se** | | **Z** | | **p** | | | **CI Lower Bound** | | | **CI Upper Bound** | | |  |
| --- | --- | --- | --- | --- | --- | --- | --- | --- | --- | --- | --- | --- | --- | --- | --- | --- | --- |
| Intercept |  | 0.275 |  | 0.451 |  | 0.609 |  | | 0.542 |  | | -0.609 |  | | 1.158 |  | |
| Gender (%male)^1^ |  | 0.148 |  | 0.578 |  | 0.255 |  | | 0.799 |  | | -0.985 |  | | 1.280 |  | |
| Intercept |  | 0.905 |  | 0.472 |  | 1.918 |  | | 0.055 |  | | -0.020 |  | | 1.831 |  | |
| Age category^2^ |  | -0.266 |  | 0.316 |  | -0.840 |  | | 0.401 |  | | -0.885 |  | | 0.354 |  | |
| Intercept |  | -0.218 |  | 0.200 |  | -1.09 |  | | 0.277 |  | | -0.610 |  | | 0.175 |  | |
| Age category^3^ |  | 0.605 |  | 0.129 |  | 4.69 |  | | <.001 |  | | 0.352 |  | | 0.858 |  | |
| Intercept |  | -- |  | -- |  | -- |  | | -- |  | | -- |  | | -- |  | |
| Intervention modality^4^ |  | -- |  | -- |  | -- |  | | -- |  | | -- |  | | -- |  | |
| Intercept |  | 0.372 |  | 0.091 |  | 4.11 |  | | <.001 |  | | 0.195 |  | | 0.550 |  | |
| Time |  | 0.002 |  | <.0001 |  | 4.69 |  | | <.001 |  | | 0.001 |  | | 0.002 |  | |
| Note. Tau² Estimator: Restricted Maximum-Likelihood; ^1^*k* = 4 since gender was not always reported in the included studies; ^2^ Reference group: 1 = young adolescents; ^3^Reference group: 1 = caregivers; ^4^All included interventions were provided in-person | | | | | | | | | | | | | | | | |  |

| Supplement Table 16 : Publication Bias Assessment | | | | | |
| --- | --- | --- | --- | --- | --- |
| **Test Name** | | **value** | | **p** | |
| Fail-Safe N |  | 121.00 |  | < .001 |  |
| Begg and Mazumdar Rank Correlation |  | -0.400 |  | 0.483 |  |
| Egger's Regression |  | -1.585 |  | 0.113 |  |
| Trim and Fill Number of Studies |  | 2.000 |  | . |  |
| Note. Fail-safe N Calculation Using the Rosenthal Approach | | | | | |

Supplement Figure 3 : Funnel Plot


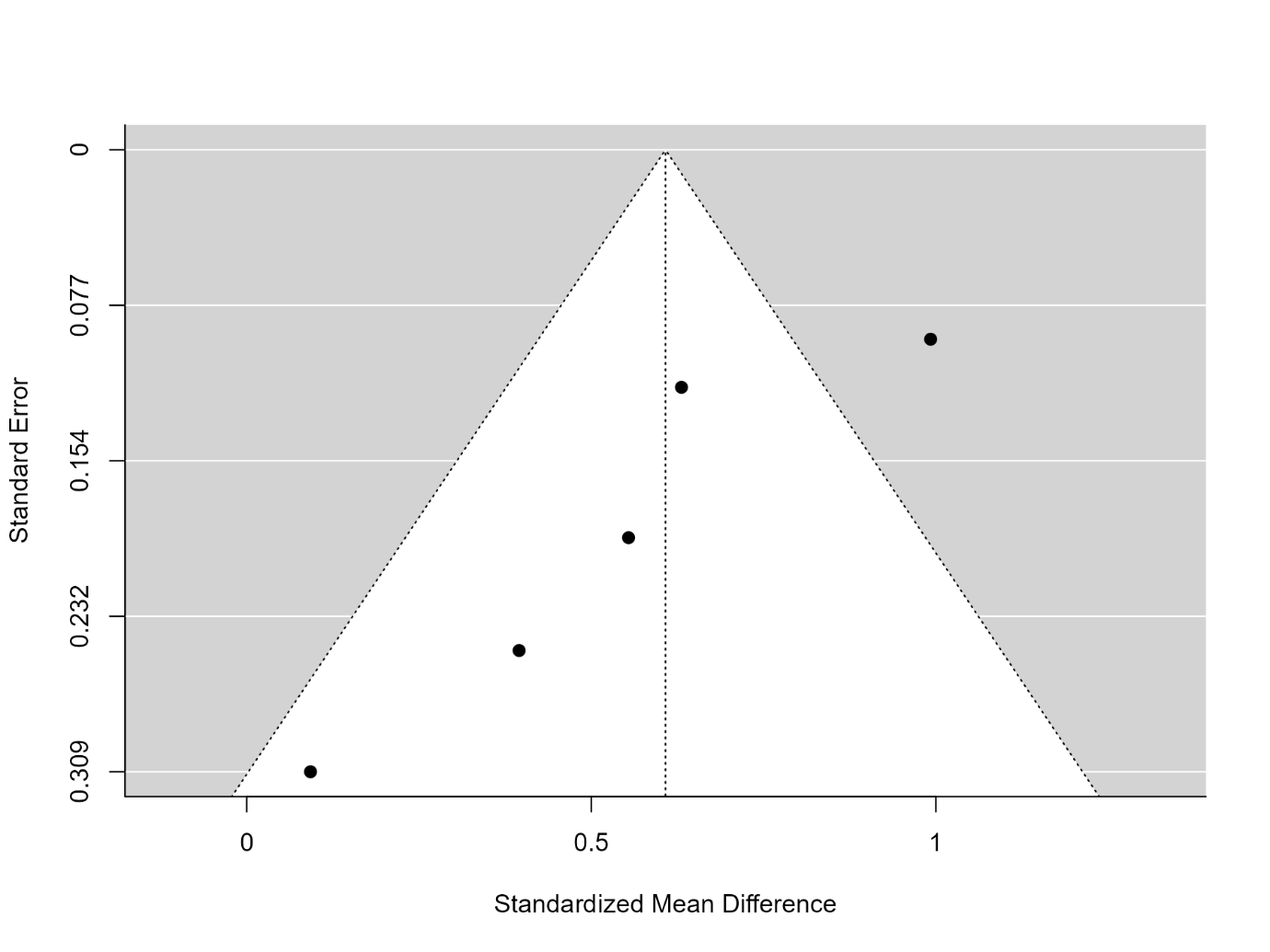


# Supplement: References list of included article (n=22)

(Caron et al., 2018; Cranmer et al., 2021; Cusimano et al., 2014; Eagles et al., 2016; Echlin et al., 2014; Elliott & Leary, 2016; Glang et al., 2015; Hunt, 2015; Kantorski et al., 2020; Kroshus et al., 2015, 2023; Kurowski et al., 2015; Labiste et al., 2021; Macdonald & Hauber, 2016; Manasse-Cohick & Shapley, 2014; Sullivan et al., 2018, 2023; Tallapragada & Cranmer, 2022; Wallace et al., 2019; Warmath & Winterstein, 2020; Wicklund et al., 2021; Zhou et al., 2022)

Caron, J. G., Rathwell, S., Delaney, J. S., Johnston, K. M., Ptito, A., & Bloom, G. A. (2018). Development, implementation and assessment of a concussion education programme for high school student-athletes. *Journal of Sports Sciences*, *36*(1), 48–55. https://doi.org/10.1080/02640414.2017.1280180

Cranmer, G., Rey, R., & Tallapragada, M. (2021). Exploring the role of parents’ sport orientations in the efficacy of concussion intervention materials. *Communication Research Reports*, *38*(4), 250–261. Communication & Mass Media Complete. https://doi.org/10.1080/08824096.2021.1936480

Cusimano, M. D., Chipman, M., Donnelly, P., & Hutchison, M. G. (2014). Effectiveness of an educational video on concussion knowledge in minor league hockey players: A cluster randomised controlled trial. *British Journal of Sports Medicine*, *48*(2), 141–146. https://doi.org/10.1136/bjsports-2012-091660

Eagles, M. E., Bradbury-Squires, D. J., Powell, M. F., Murphy, J. R., Campbell, G. D., & Maroun, F. B. (2016). The Impact of a Concussion-U Educational Program on Knowledge of and Attitudes about Concussion. *The Canadian Journal of Neurological Sciences. Le Journal Canadien Des Sciences Neurologiques*, *43*(5), 659–664. https://doi.org/10.1017/cjn.2016.263

Echlin, P. S., Johnson, A. M., Holmes, J. D., Tichenoff, A., Gray, S., Gatavackas, H., Walsh, J., Middlebro, T., Blignaut, A., MacIntyre, M., Anderson, C., Fredman, E., Mayinger, M., Skopelja, E. N., Sasaki, T., Bouix, S., Pasternak, O., Helmer, K. G., Koerte, I. K., … Forwell, L. A. (2014). The Sport Concussion Education Project. A brief report on an educational initiative: From concept to curriculum. *Journal of Neurosurgery*, *121*(6), 1331–1336. https://doi.org/10.3171/2014.8.JNS132804

Elliott, R., & Leary, L. (2016). Pediatric Sport-Related Concussion Education: Effectiveness and Long-Term, Retention of the Head Safety Youth Sports (HSYS) Program for Youth Athletes, Ages 11-16. *NEUROLOGY*, *86*.

Glang, A. E., Koester, M. C., Chesnutt, J. C., Gioia, G. A., McAvoy, K., Marshall, S., & Gau, J. M. (2015). The effectiveness of a web-based resource in improving postconcussion management in high schools. *The Journal of Adolescent Health : Official Publication of the Society for Adolescent Medicine*, *56*(1), 91–97. https://doi.org/10.1016/j.jadohealth.2014.08.011

Hunt, T. N. (2015). Video Educational Intervention Improves Reporting of Concussion and Symptom Recognition. *Athletic Training Education Journal*, *10*(1), 65–74. https://doi.org/10.4085/100165

Kantorski, B., Commisso, D. R., Sanford-Dolly, C. W., & Pollock, J. A. (2020). The Use of a Mobile Application to Teach Concussion-Related Health Knowledge. *Journal of STEM Outreach*, *3*(1). https://doi.org/10.15695/jstem/v3i1.05

Kroshus, E., Baugh, C. M., Hawrilenko, M., & Daneshvar, D. H. (2015). Pilot randomized evaluation of publically available concussion education materials: Evidence of a possible negative effect. *Health Education & Behavior : The Official Publication of the Society for Public Health Education*, *42*(2), 153–162. https://doi.org/10.1177/1090198114543011

Kroshus, E., Chrisman, S. P. D., Glang, A., Hunt, T., Hays, R., Lowry, S., Peterson, A., Garrett, K., Ramshaw, D., Hafferty, K., Kinney, E., Manzueta, M., Steiner, M. K., Bollinger, B. J., Chiampas, G., & Rivara, F. P. (2023). Concussion education for youth athletes using Pre-Game Safety Huddles: A cluster-randomised controlled trial. *Injury Prevention : Journal of the International Society for Child and Adolescent Injury Prevention*, *29*(1), 22–28. https://doi.org/10.1136/ip-2022-044665

Kurowski, B. G., Pomerantz, W. J., Schaiper, C., Ho, M., & Gittelman, M. A. (2015). Impact of preseason concussion education on knowledge, attitudes, and behaviors of high school athletes. *The Journal of Trauma and Acute Care Surgery*, *79*(3 Suppl 1), S21-28. https://doi.org/10.1097/TA.0000000000000675

Labiste, C. C., McElroy, E., Chaniotakis, S., Duong, N., & Haffizulla, F. (2021). Effectiveness of PitchSafe on Knowledge and Attitude of Baseball-Related Concussion. *Cureus*, *13*(4), e14732. https://doi.org/10.7759/cureus.14732

Macdonald, I., & Hauber, R. (2016). Educating Parents on Sports-Related Concussions. *The Journal of Neuroscience Nursing : Journal of the American Association of Neuroscience Nurses*, *48*(6), 297–302. https://doi.org/10.1097/JNN.0000000000000212

Manasse-Cohick, N. J., & Shapley, K. L. (2014). Concussion Education for High School Football Players: A Pilot Study. *Communication Disorders Quarterly*, *35*(3), 182–185. https://doi.org/10.1177/1525740113506605

Sullivan, L., McKenzie, L. B., Roberts, K., Recker, R., Schwebel, D. C., Pommering, T., & Yang, J. (2023). A Virtual Reality App Intervention to Improve Concussion Recognition and Reporting in Athletes Aged 9 to 12 Years: Development and Pilot Testing. *JMIR Formative Research*, *7*, e43015. https://doi.org/10.2196/43015

Sullivan, L., Pursell, L., & Molcho, M. (2018). Evaluation of a theory-based concussion education program for secondary school student-athletes in Ireland. *Health Education Research*, *33*(6), 492–504. https://doi.org/10.1093/her/cyy034

Tallapragada, M., & Cranmer, G. (2022). Media Narratives About Concussions: Effects on Parents’ Intention to Inform Their Children About Concussions. *COMMUNICATION & SPORT*, *10*(3), 517–540. https://doi.org/10.1177/2167479520944549

Wallace, J., Covassin, T., & Beidler, E. (2019). Concussion Bingo: Taking an active learning approach to concussion education with vulnerable populations. *Health Education Journal*, *78*(3), 315–327. https://doi.org/10.1177/0017896918806935

Warmath, D., & Winterstein, A. P. (2020). A Social-Marketing Intervention and Concussion-Reporting Beliefs. *Journal of Athletic Training*, *55*(10), 1035–1045. https://doi.org/10.4085/1062-6050-242-19

Wicklund, A., Roy, A., & Coatsworth, J. D. (2021). Providing a Medical Definition of Concussion: Can a Simple Intervention Improve Self-Reported Concussion History in Youth Athletes? *Clinical Journal of Sport Medicine: Official Journal of the Canadian Academy of Sport Medicine*, *31*(6), e467–e469. https://doi.org/10.1097/JSM.0000000000000813

Zhou, H., Ledsky, R., Sarmiento, K., DePadilla, L., Kresnow, M.-J., & Kroshus, E. (2022). Parent–Child communication about concussion: What role can the Centers for Disease Control and Prevention’s HEADS UP concussion in youth sports handouts play? *Brain Injury*, *36*(9), 1133–1139. Psychology and Behavioral Sciences Collection.
